# Phosphomannosylation and the Functional Analysis of the Extended *Candida albicans MNN4*-Like Gene Family

**DOI:** 10.3389/fmicb.2017.02156

**Published:** 2017-11-06

**Authors:** Roberto J. González-Hernández, Kai Jin, Marco J. Hernández-Chávez, Diana F. Díaz-Jiménez, Elías Trujillo-Esquivel, Diana M. Clavijo-Giraldo, Alma K. Tamez-Castrellón, Bernardo Franco, Neil A. R. Gow, Héctor M. Mora-Montes

**Affiliations:** ^1^División de Ciencias Naturales y Exactas, Departamento de Biología, Universidad de Guanajuato, Guanajuato, Mexico; ^2^Aberdeen Fungal Group, Institute of Medical Sciences, University of Aberdeen, Aberdeen, United Kingdom; ^3^School of Life Sciences, Chongqing University, Chongqing, China; ^4^Centro de Investigación y de Estudios Avanzados del Instituto Politécnico Nacional, Guanajuato, Mexico

**Keywords:** cell wall, phosphomannosylation, *Candida albicans*, phagocytosis, phosphomannosyltransferase, CRISPR-Cas9 system, mini-Ura-blaster

## Abstract

Phosphomannosylation is a modification of cell wall proteins that occurs in some species of yeast-like organisms, including the human pathogen *Candida albicans*. These modified mannans confer a negative charge to the wall, which is important for the interactions with phagocytic cells of the immune systems and cationic antimicrobial peptides. In *Saccharomyces cerevisiae*, the synthesis of phosphomannan relies on two enzymes, the phosphomannosyltransferase Ktr6 and its positive regulator Mnn4. However, in *C. albicans*, at least three phosphomannosyltransferases, Mnn4, Mnt3 and Mnt5, participate in the addition of phosphomannan. In addition to *MNN4, C. albicans* has a *MNN4*-like gene family composed of seven other homologous members that have no known function. Here, using the classical mini-Ura-blaster approach and the new gene knockout CRISPR-Cas9 system for gene disruption, we generated mutants lacking single and multiple genes of the *MNN4* family; and demonstrate that, although Mnn4 has a major impact on the phosphomannan content, *MNN42* was also required for full protein phosphomannosylation. The reintroduction of *MNN41, MNN42, MNN46*, or *MNN47* in a genetic background lacking *MNN4* partially restored the phenotype associated with the *mnn4*Δ null mutant, suggesting that there is partial redundancy of function between some family members and that the dominant effect of *MNN4* over other genes could be due to its relative abundance within the cell. We observed that additional copies of alleles number of any of the other family members, with the exception of *MNN46*, restored the phosphomannan content in cells lacking both *MNT3* and *MNT5*. We, therefore, suggest that phosphomannosylation is achieved by three groups of proteins: [i] enzymes solely activated by Mnn4, [ii] enzymes activated by the dual action of Mnn4 and any of the products of other *MNN4*-like genes, with exception of *MNN46*, and [iii] activation of Mnt3 and Mnt5 by Mnn4 and Mnn46. Therefore, although the *MNN4*-like genes have the potential to functionally redundant with Mnn4, they apparently do not play a major role in cell wall mannosylation under most *in vitro* growth conditions. In addition, our phenotypic analyses indicate that several members of this gene family influence the ability of macrophages to phagocytose *C. albicans* cells.

## Introduction

Infections caused by members of the *Candida* genus can result in both superficial and deep tissue invasion—the latter frequently being associated with immunocompromised patients resulting in high morbidity and mortality rates (Brown et al., 2012). Of the 190 species classified within the *Candida* genus, *Candida albicans* is the most frequent agent of candidiasis, although other *Candida* species are increasingly becoming a major cause of concern (Sanguinetti et al., 2015).

The *C. albicans* cell wall plays a key role in cellular fitness and as a molecular scaffold to which several virulence-related proteins are attached (Mora-Montes et al., 2009; Gow and Hube, 2012; Erwig and Gow, 2016). Indeed, mutant strains with defects in the synthesis of cell wall components often display virulence attenuation and alterations in immune recognition (Bates et al., 2005, 2006, 2013; Munro et al., 2005; Prill et al., 2005; Mora-Montes et al., 2007, 2010; Hall et al., 2013; Courjol et al., 2015). The *Candida* cell wall is composed of an inner core of chitin, β1, 3- and β1, 6-glucans, and an outer layer of highly glycosylated proteins that are rich in various classes of mannoligosaccharides (mannoproteins) (Klis et al., 2001; Hall and Gow, 2013). These glycoproteins have high molecular weight-branched mannose oligosaccharides linked to Asp (*N*-liked mannans) and shorter linear glycans attached to Ser/Thr (*O*-linked mannans) residues (Mora-Montes et al., 2009; Hall and Gow, 2013). In yeast-like organisms, such as *Saccharomyces cerevisiae* or *C. albicans*, both types of mannans can be modified by ether-linked mannosylphosphate (phosphomannan) (Mora-Montes et al., 2009; Orlean, 2012). The *N*-linked mannan has an oligosaccharide core synthesized in the endoplasmic reticulum, which is modified by Golgi-resident mannosyltransferases that elaborate the α1, 6-polymannose backbone, and lateral branched mannooligosaccharides that are α1, 2-, α1, 3- or β1, 2-linked (Martinez-Duncker et al., 2014). The *O*-linked mannans of up to seven units are composed predominantly of α1, 2-mannose sugars (Munro et al., 2005; Diaz-Jimenez et al., 2012). This phosphomannan moiety is believed to participate in the regulation of nucleotide transport across the Golgi membrane, in the cross-linking between cell wall proteins and glucan, and in stress regulation during stationary growth phase or under conditions of drought or high osmolality (Jigami and Odani, 1999). Charge neutral *C. albicans* mutant cells that lack cell wall phosphomannan showed increased resistance to the inhibitory effect of the cationic peptide DsS3(1-16) (Harris et al., 2009), and were less quickly phagocytosed by primary macrophages and macrophage cell lines (McKenzie et al., 2010; Lewis et al., 2012; Bain et al., 2014). These observations underscore the importance of this cell wall component during the host-fungus interaction.

Phosphomannan synthesis has been characterized in most detail in *S. cerevisiae*. The Golgi-resident Mnn6/Ktr6 protein is the sole phosphomannosyltransferase in this organism and responsible for the addition of phosphomannan to both *N*-linked and *O*-linked mannans (Wang et al., 1997). Although there has been no enzyme activity associated with Mnn4, this protein is presumed to be a positive regulator of the phosphomannosyltransferase, because overexpression or disruption of *MNN4* positively and negatively affected the cell wall phosphomannan content, respectively (Odani et al., 1996; Jigami and Odani, 1999). Recently, Mnn14 has been also involved in the phosphomannosylation of *S. cerevisiae N*-linked mannans (Kim et al., 2017). This gene is a paralog of *MNN4*, and these proteins showed functional redundancy in addition of mannosylphosphate to the *N*-linked mannan core (Kim et al., 2017). In *C. albicans, MNT3* and *MNT5* encode for functional orthologs of *S. cerevisiae MNN6/KTR6*, with redundant phosphomannosyltransferase activity, and are involved in the elaboration of about 50% of the cell wall phosphomannan (Mora-Montes et al., 2010). This organism also contains a functional ortholog of *S. cerevisiae MNN4*, and the cell wall phosphomannan is barely detected in a *C. albicans mnn4*Δ null mutant (Hobson et al., 2004). In contrast to *S. cerevisiae*, the *C. albicans* phosphomannan moiety appears to function as a molecular scaffold for the addition of linear β1, 2-oligosaccharides of up to 14 mannose residues (Hobson et al., 2004; Mora-Montes et al., 2009). This participates in establishing the hydrophobic properties of the cell (Singleton et al., 2005). Interestingly, in *C. albicans* there is an extended *MNN4*-like gene family that is composed of seven additional *MNN4*-like genes of unknown function, named *MNN41, MNN42, MNN43, MNN44, MNN45, MNN46*, and *MNN47* (Butler et al., 2009).

To investigate the function of the members of this gene family, we disrupted *MNN4* along with the seven other orthologs and characterized their ability to bind Alcian Blue. We found that, aside from deletion of *MNN4*, only disruption of *MNN42* was capable of reducing the gross cell wall phosphomannan content. However, overexpression of a range of *MNN4*-like genes in genetic backgrounds lacking either *MNN4* or *MNT3* and *MNT5* showed that these genes could complement the mutant phenotype to some extent. These data suggest that all the *MNN4*-like genes family are capable of participating in the elaboration of *C. albicans* cell wall phosphomannan under permissive conditions.

## Materials and methods

### Strains and culturing conditions

Strains used in this work are listed in Table 1. Cells were growth in YPD medium (1% [w/v] yeast extract, 2% [w/v] gelatin peptone, 2% [w/v] dextrose) at 28°C and 200 rpm. When solid medium was required, 2% (w/v) agar was added. Yeast transformants were selected in SD medium (0.76% [w/v] yeast nitrogen base with ammonium sulfate without amino acids, 2% [w/v] dextrose and 0.077% [w/v] complete supplement mixture minus uracil] with 50 μg/mL uridine when required. For selection of transformants using the CRISPR-Cas9 strategy, cells were grown on YPD agar supplemented with 200 μg/mL nourseothricin (ClonNAT, WERNER BioAgents, Jena, Germany).

**Table 1:** Strains used in this work.

**Strain**	**Genotype**	**References**
CAI4	*ura3*Δ::*imm*434*/ura3*Δ::*imm*434	Fonzi and Irwin, 1993
BWP17	*ura3*Δ::*imm434/ura3*Δ::*imm434, his1*Δ::*hisG/his1*Δ::*hisG, arg4*Δ::*hisG/arg4*Δ::*hisG*	Wilson et al., 1999
NGY152	As CAI4, but *RPSI*/*rps1*Δ::Clp10	Brand et al., 2004
CDH7	As CAI4, but *mnn4*Δ::*hisG/mnn4*Δ::*hisG*	Hobson et al., 2004
CDH15	As CDH7, but *RPSI*/*rps1*Δ::Clp10	Hobson et al., 2004
NGY522	As CAI4, but *mnt5*Δ::*higG*/*mnt5*Δ::*hisG; mnt3*Δ::*dp1200/mnt3*Δ::*dp1200*	Mora-Montes et al., 2010
NGY1227	As NGY522, but *RPS1/rps1*Δ::CIp10	Mora-Montes et al., 2010
HMY189	As CAI4, but *mnn41*Δ::*dp1200/mnn41*Δ::*dp1200, RPS1/rps1*Δ:: Clp10	This work
HMY81	As CAI4, but *mnn42*Δ::*dp1200/mnn42*Δ::*dp1200, RPS1/rps1*Δ:: Clp10	This work
HMY84	As CAI4, but *mnn42*Δ::*dp1200/mnn42*Δ::*dp1200, RPS1/rps1*Δ:: Clp10-*MNN42*	This work
HMY190	As CAI4, but *mnn43*Δ::*dp1200/mnn43*Δ::*dp1200, RPS1/rps1*Δ:: Clp10	This work
HMY191	As CAI4, but *mnn44*Δ::*dp1200/mnn44*Δ::*dp1200, RPS1/rps1*Δ:: Clp10	This work
HMY192	As CAI4, but *mnn45*Δ::*dp1200/mnn45*Δ::*dp1200, RPS1/rps1*Δ:: Clp10	This work
HMY193	As CAI4, but *mnn46*Δ::*dp1200/mnn46*Δ::*dp1200, RPS1/rps1*Δ:: Clp10	This work
HMY194	As CAI4, but *mnn47*Δ::*dp1200/mnn47*Δ::*dp1200, RPS1/rps1*Δ:: Clp10	This work
HMY195	As CAI4, but *mnn41*Δ::*dp1200/mnn41*Δ::*dp1200, mnn47*Δ::*dp1200/mnn47*Δ::*dp1200, RPS1/rps1*Δ:: Clp10	This work
HMY196	As CAI4, but *mnn41*Δ::*dp1200/mnn41*Δ::*dp1200, mnn47*Δ::*dp1200/mnn47*Δ::*dp1200, RPS1/rps1*Δ:: Clp10-*MNN41*	This work
HMY197	As CAI4, but *mnn41*Δ::*dp1200/mnn41*Δ::*dp1200, mnn47*Δ::*dp1200/mnn47*Δ::*dp1200, RPS1/rps1*Δ:: Clp10-*MNN47*	This work
HMY198	As CAI4, but *mnn41*Δ::*dp1200/mnn41*Δ::*dp1200, mnn42*Δ::*dp1200/mnn42*Δ::*dp1200, mnn47*Δ::*dp1200/mnn47*Δ::*dp1200, RPS1/rps1*Δ:: Clp10	This work
HMY199	As CAI4, but *mnn41*Δ::*dp1200/mnn41*Δ::*dp1200, mnn42*Δ::*dp1200/mnn42*Δ::*dp1200, mnn47*Δ::*dp1200/mnn47*Δ::*dp1200, RPS1/rps1*Δ:: Clp10-*MNN41*	This work
HMY200	As CAI4, but *mnn41*Δ::*dp1200/mnn41*Δ::*dp1200, mnn42*Δ::*dp1200/mnn42*Δ::*dp1200, mnn47*Δ::*dp1200/mnn47*Δ::*dp1200, RPS1/rps1*Δ:: Clp10-*MNN42*	This work
HMY201	As CAI4, but *mnn41*Δ::*dp1200/mnn41*Δ::*dp1200, mnn42*Δ::*dp1200/mnn42*Δ::*dp1200, mnn47*Δ::*dp1200/mnn47*Δ::*dp1200, RPS1/rps1*Δ:: Clp10-*MNN47*	This work
HMY202	As CAI4, but *mnn43*Δ::*dp1200/mnn43*Δ::*dp1200, mnn44*Δ::*dp1200/mnn44*Δ::*dp1200, mnn45*Δ::*dp1200/mnn45*Δ::*dp1200, mnn46*Δ::*dp1200/mnn46*Δ::*dp1200, RPS1/rps1*Δ:: Clp10	This work
NGY648	As BWP17, but *mnn4*Δ/*mnn4*Δ, *mnn41*Δ/*mnn41*Δ, *mnn42*Δ/*mnn42*Δ, *mnn43*Δ/*mnn43*Δ, *mnn44*Δ/*mnn44*Δ, *mnn45*Δ/*mnn45*Δ, *mnn46*Δ/*mnn46*Δ, *mnn47*Δ/*mnn47*Δ	This work
HMY95	As CDH7, but *RPSI*/*rps1*Δ::Clp10-*MNN41*	This work
HMY92	As CDH7, but *RPSI*/*rps1*Δ::Clp10-*MNN42*	This work
HMY104	As CDH7, but *RPSI*/*rps1*Δ::Clp10-*MNN43*	This work
HMY167	As CDH7, but *RPSI*/*rps1*Δ::Clp10-*MNN44*	This work
HMY168	As CDH7, but *RPSI*/*rps1*Δ::Clp10-*MNN45*	This work
HMY164	As CDH7, but *RPSI*/*rps1*Δ::Clp10-*MNN46*	This work
HMY123	As CDH7, but *RPSI*/*rps1*Δ::Clp10-*MNN47*	This work
HMY96	As NGY522, but *RPS1/rps1*Δ::CIp10-*MNN41*	This work
HMY87	As NGY522, but *RPS1/rps1*Δ::CIp10-*MNN42*	This work
HMY103	As NGY522, but *RPS1/rps1*Δ::CIp10-*MNN43*	This work
HMY165	As NGY522, but *RPS1/rps1*Δ::CIp10-*MNN44*	This work
HMY159	As NGY522, but *RPS1/rps1*Δ::CIp10-*MNN45*	This work
HMY166	As NGY522, but *RPS1/rps1*Δ::CIp10-*MNN46*	This work
HMY124	As NGY522, but *RPS1/rps1*Δ::CIp10-*MNN47*	This work

### Construction of null mutants

The mini-ura-blaster technique was used for gene disruption as follows. Specific primer pairs for each gene, containing complementary sequences to the 5′- and 3′- regions of the target ORF (see Supplementary Material, Table S1) were used to amplify by PCR the disruption cassette from the pDDB57 plasmid (Wilson et al., 2000). The strain CAI4, a Ura^−^ mutant derived from the clinical isolated SC5314 (Fonzi and Irwin, 1993), was transformed sequentially with the disruption cassettes and the *URA3* marker recycled by growing transformants on SC medium supplemented with 1 mg/mL 5-fluoroorotic acid and uridine. The plasmid CIp10 was used to restore *URA3* at the *RPS1* locus, as previously described (Murad et al., 2000). For gene disruption using the CRISPR-Cas9 strategy, *C. albicans* strain BWP17 (Wilson et al., 1999) was used as parental strain. Plasmid for CaCas9 Solo system (pV1200 vector) was adopted in this work (Vyas et al., 2015). Constructions of knockout vectors, marker recycling and verification of CRISPR-mutagenized loci were performed according to the previous method (Vyas et al., 2015). The oligonucleotide sequences used in this study are listed in Table S2.

### Generation of constructions to complement null mutant strains

The ORF of each *MNN4-*like gene-family, plus ~1,000 bp upstream and ~600 bp downstream were amplified by PCR using primers listed in Table S3. The PCR product was cloned into *Not*I sites of the CIp10 plasmid (Murad et al., 2000). The null mutants generated in this study, a *mnn4*Δ null strain (Hobson et al., 2004) and a double *mnt3*Δ, *mnt5*Δ null strain were transformed with the constructions generated (see Table 1). Before transformation, constructions were digested with *Stu*I, and confirmation of plasmid insertion into the *RPS1* locus was performed by PCR.

### Expression analysis

Total RNA was isolated from yeast cells using TRIzol (Invitrogen), and further purified with the kit RNeasy (Qiagen), following the manufacture's instruction. The cDNA was synthesized using the SuperScript system (Invitrogen). Following synthesis, cDNA purification was conducted after RNA degradation using adsorption chromatography, as described (Trujillo-Esquivel et al., 2016). Absence of genomic DNA in the cDNA preparations was confirmed by amplification of the *ACT1* gene which contains an intron of 658 bp (data not shown).

All primer pairs used in qPCR reactions are listed in Table S4. The reaction mixtures were prepared using the SYBR® Green PCR Master Mix (Thermo Fisher Scientific) and analyzed in a StepOnePlus Real-Time PCR System (Applied Biosystems). All reactions produced a single amplicon, with a uniform melting curve, as determined by the dissociation profile of the products. Relative quantification was determined by calculating 2^−ΔΔCT^ (Livak and Schmittgen, 2001). Expression data were normalized using *RPP2B*, a housekeeping gene previously used as a control in expression assays (Nailis et al., 2006).

### Alcian blue binding assays

The phosphomannan content was determined by measuring the binding of the cationic dye Alcian Blue to the cell surface, as previously described (Hobson et al., 2004). Briefly, cells in exponential growth phase were collected, washed twice with deionized water and adjusted to an OD_600_ of 0.2. Aliquots of 1 mL were pelleted, and cells suspended in 1 mL of 30 μg/mL Alcian Blue (in 0.02 M HCl) and incubated at room temperature for 15 min. The cell suspension was then centrifuged and the supernatant saved and used to quantify the content of Alcian Blue absorbance at 620 nm. The concentration of the free (non-bound) dye was then determined and used to calculate the amount of Alcian Blue bound to cells as described (Hobson et al., 2004).

### Phenotypic characterization of null mutants

Cells from overnight cultures grown in YPD at 28°C and 200 rpm were used to inoculate fresh YPD broth and cell growth was monitored every 30 min by absorbance at 600 nm. Yeast-hypha dimorphism was evaluated by incubating 5 × 10^6^ cells/mL in RPMI 1640 (Sigma) supplemented with 10% (v/v) fetal bovine serum (Sigma) for 4 h at 37°C and 200 rpm. Cell preparations were inspected by bright-field microscopy to evaluate the percentage of yeast cells, pseudohyphae and hyphae. Two hundred cells were counted per strain. Alternatively, hypha formation was stimulated by growing cells on solid Spider medium (Liu et al., 1994). The robustness of the cell wall was tested by assessing the sensitivity of yeast cells to a range of cell wall perturbing agents. Cells in the exponential growth phase were collected, washed twice with deionized water, adjusted to an OD_600_ of 0.05 and seeded into a 96-well plate containing YPD plus doubling dilutions of: SDS, Calcofluor White, Congo Red, and hygromycin B. Plates were incubated at 28°C for 24 h and then read at OD_600_. The highest concentrations tested for each agent were: 0.25% (v/v) SDS, 100 μg/mL Calcofluor White, 100 μg/mL Congo Red, and 500 μg/mL hygromycin B.

### Phagocytosis analysis

The RAW 264.7 (ATCC® TIB-71™) murine cell line was cultured in DMEM media (Sigma), supplemented with 10% (v/v) fetal bovine, at 37°C and 5% (v/v) CO_2._ After reaching 90% confluence, cells were detached using trypsin (Sigma) in DMEM medium and sub-seeded into 6-well plates for further growth. Cells were again harvested by trypsin detachment and the concentration adjusted to 2 × 10^5^ cells/mL in DMEM medium. Yeast cells were grown in YPD at 28°C with reciprocal shaking at 200 rpm until they reached exponential growth phase and were then washed twice with PBS, and labeled simultaneously with Acridine Orange (1 mg/mL, Sigma) as reported (Abrams et al., 1983). The fungal cells were washed twice with PBS and resuspended at a cell density of 1 × 10^7^ cell/mL. Phagocytosis assays were carried out in a total volume of 800 μL of fresh DMEM, in 6-well plates. The macrophage:yeast cell ratio was set at 1:3, and interactions were incubated for 2.5 h at 37°C under a CO_2_ atmosphere. Macrophages were washed once with PBS and detached from plates using trypsin. Cells were washed twice with PBS by centrifuging at 200 × g for 10 min at 4°C, and resuspended in PBS containing 1.25 mg/mL Trypan Blue as an external fluorescence quencher, as described previously (Santos et al., 2015). Macrophages were kept on ice until they were analyzed by flow cytometry.

Flow cytometry was performed in a MoFlo XDP system (Beckman Coulter) collecting 50,000 events that were singlet events, and then gated for macrophage cells. Fluorescence was recovered from the compensated FL1 (green) and FL3 (red) channels using macrophage cells without any labeling. Phagocytosis of yeast cells was assessed by counts in the green (recently phagocytosed cells) and red (cells within acidified phagolysosomes) fluorescence channels.

### Statistical analysis

Statistical analyses were performed using GraphPad Prism 7 software. All the experiments were performed with three biological replicates in duplicate. Data represent cumulative results of all experiments performed and are shown as means ± S.D. The Mann-Whitney *U-*test was used to establish statistical significance, which was set at *P* < 0.05.

## Results

### Disruption of *C. albicans MNN4* family genes

**Figure 1:**
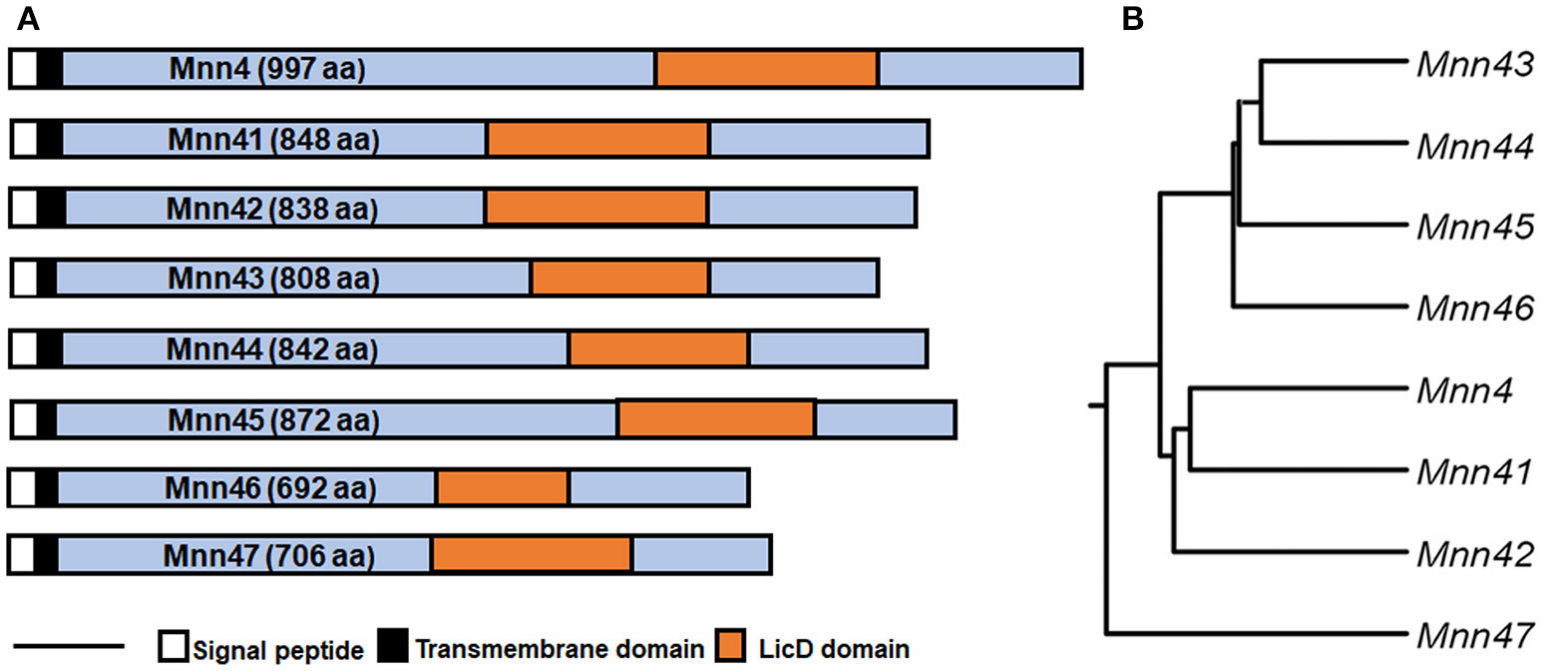
Phylogenetic tree of members of the *C. albicans MNN4*-like gene family. **(A)** The eight members of the *MNN4*-like gene family, indicating relevant features of the primary amino acid sequences. The scale bar represents 100 amino acids **(B)**, Dendrogram generated using the Neighbour-joining tree without distance correction algorithm defining three clades, (i) Mnn43, Mnn44, Mnn45 and Mnn46; (ii) Mnn4, Mnn41 and Mnn42; and (iii) Mnn47 as a sole outlier. The amino acid sequences were retrieved from http://www.candidagenome.org, archived under the following systematic names: *MNN4* (C4_06540W_A), *MNN41* (C2_03710W_A), *MNN42* (C2_03690C_A), *MNN43* (C1_02670C_A), *MNN44* (C1_02680C_A), *MNN45* (C6_02830W_A), *MNN46* (C4_06990W_A), *MNN47* (C1_09130W_A).

The *C. albicans MNN4* gene has been previously characterized, and encodes a protein of 997 amino acids, with a putative signal peptide, a transmembrane domain, and an N-terminal region of lysine/glutamic acid repeats that is essential for enzyme activity (Hobson et al., 2004). Near the C-terminal end is a domain that is common to members of the LicD family of proteins, which are involved in phosphocholine metabolism (Zhang et al., 1999). This domain is present in proteins that belong to the nucleotidyltransferase superfamily, which includes enzymes that catalyze the transfer of nucleoside monophosphate to the hydroxyl group of an acceptor, using a nucleoside triphosphate as the donor (Kuchta et al., 2009). Bioinformatics analyses of putative encoded products of *MNN4*-like genes indicated that all of them have the LicD domain, a putative signal peptide, and a transmembrane domain close to the N-terminus (Figure 1A). These analyses did not identify lysine/glutamic acid repeats in the other *MNN4* genes, but some revealed the presence of glutamine repeats, of 2–4 amino acids: For Mnn45 these repeats were closer to the N-terminus, whereas in Mnn41, Mnn42, and Mnn47 they were closer to the C- terminus. No glutamine repeats were identified in Mnn43, Mnn44, and Mnn46 (data not shown). All the seven family members had similarity scores of ~50% to Mnn4, varying between 43% for Mnn41 and Mnn45 to 58% for Mnn42 and Mnn47 (Table 2). The putative amino acid sequences of the *MNN4*-like genes were used to generate a phylogenetic tree (Figure 1B), using the “Neighbor-joining tree without distance correction” algorithm (Sievers et al., 2011). This analysis showed three main groups: one composed of Mnn43, Mnn44, Mnn45, and Mnn46; the second of Mnn4, Mnn41, and Mnn42; while Mnn47 was isolated as a single outlier (Figure 1B). This dendrogram was used to design subsequent sequential gene disruption strategies.

**Table 2:** Comparison of the putative protein sequence of the *C. albicans MNN4*-like gene family members.

	**Mnn4**	**Mnn41**	**Mnn42**	**Mnn43**	**Mnn44**	**Mnn45**	**Mnn46**	**Mnn47**
Mnn4	100/100^*^	40/56	41/61	32/50	31/49	34/51	35/52	39/57
Mnn41	40/56	100/100	34/51	29/44	30/48	29/43	28/46	29/46
Mnn42	41/61	34/51	100/100	27/46	26/46	31/52	31/49	35/58
Mnn43	32/50	29/44	27/46	100/100	36/56	33/52	31/48	26/46
Mnn44	34/51	30/48	26/46	36/56	100/100	34/51	32/49	30/54
Mnn45	32/49	29/43	31/52	33/52	34/51	100/100	32/48	35/55
Mnn46	35/52	28/46	31/49	31/48	32/49	32/48	100/100	27/46
Mnn47	39/57	29/46	35/58	26/46	30/54	35/55	27/46	100/100

* *Numbers represent percentage of identity and similarity, respectively*.

The *C. albicans* strain CAI4, a Ura^−^ derived strain from the clinical isolate SC5314 (Fonzi and Irwin, 1993), was used as genetic background for the generation of single null mutants, using the mini-Ura-blaster strategy (Wilson et al., 2000). These null mutants were used as background to generate double, triple, or quadruple null mutants. To avoid problems due to ectopic expression of *URA3* (Brand et al., 2004), the resulting null mutants were transformed with the *Stu*I-linearized CIp10 plasmid (Murad et al., 2000), introducing *URA3* at the neutral *RPS1* locus. The strain CAI4 transformed with the *Stu*I-linearized CIp10 plasmid (NGY152) (Brand et al., 2004) was used as a wild-type (WT) control strain. The re-integrant control strains were constructed introducing the gene of interest at the *RPS1* locus, as described in Materials and Methods. Furthermore, to generate a mutant strain lacking all eight *MNN4*-like genes, the CRISPR-Cas9 system (Vyas et al., 2015) was used in the BWP17 genetic background (Wilson et al., 1999). None of the strains generated in this study (see Table 1) displayed changes in the growth rate, ability to undergo dimorphism, cell and colony morphologies, and the sensitivity to the cell wall perturbing agents Congo Red, Calcofluor White, SDS, and hygromycin B (data not shown). Mutants derived independently in parallel lineages in which the order of disruption of individual genes differed, all generated the same final phenotype.

**Figure 2:**
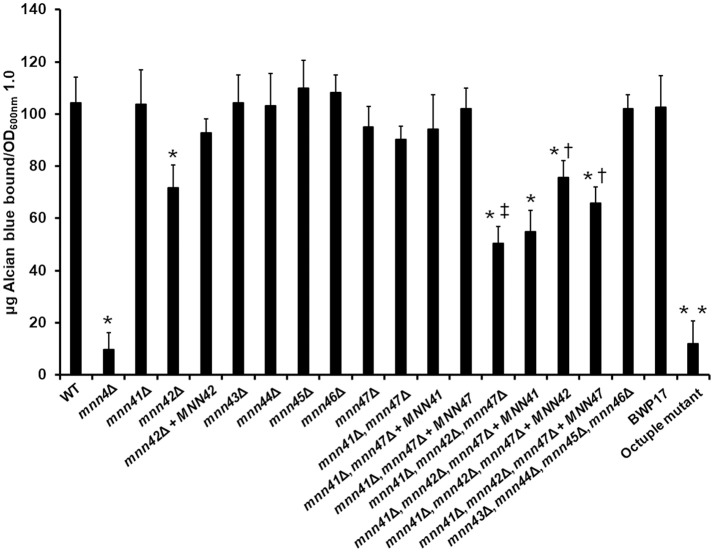
Members of the *C. albicans MNN4*-like gene family participate in cell wall phosphomannosylation. Cells were grown in YPD medium, and ability to bind Alcian Blue was measured. The strains used are: NGY152 (WT), CDH15 (*mnn4*Δ), HMY189 (*mnn41*Δ), HMY81 (*mnn42*Δ), HMY84 (*mnn42*Δ + *MNN42*), HMY190 (*mnn43*Δ), HMY191 (*mnn44*Δ), HMY192 (*mnn45*Δ), HMY193 (*mnn46*Δ), HMY194 (*mnn47*Δ), HMY195 (*mnn41*Δ, *mnn47*Δ), HMY196 (*mnn41*Δ, *mnn47*Δ + *MNN41*), HMY197 (*mnn41*Δ, *mnn47*Δ + *MNN47*), HMY198 (*mnn41*Δ, *mnn42*Δ, *mnn47*Δ), HMY199 (*mnn41*Δ, *mnn42*Δ, *mnn47*Δ + *MNN41*), HMY200 (*mnn41*Δ, *mnn42*Δ, *mnn47*Δ + *MNN42*), HMY201 (*mnn41*Δ, *mnn42*Δ, *mnn47*Δ + *MNN47*), HMY202 (*mnn43*Δ, *mnn44*Δ, *mnn45*Δ, *mnn46*Δ); BWP17, and NGY648 (octuple mutant).The data represent the means ± *SD* of three independent assays performed by triplicate. ^*^P < 0.05 when compared against the WT strain. ^†^*P* < 0.05 when compared against the *mnn41*Δ, *mnn42*Δ, *mnn47*Δ null mutant. ^‡^*P* < 0.05 for the comparison of the triple *mnn41*Δ, *mnn42*Δ, *mnn47*Δ, and the *mnn42*Δ null mutant. ^**^P < 0.05 when the octuple mutant was compared against the parental strain BWP17.

Next, we tested the ability of the single null mutants to bind Alcian Blue, a dye that specifically binds the negative charge provided by the phosphate group of phosphomannan (Hobson et al., 2004). The WT control cells efficiently bound Alcian Blue whilst the *mnn4*Δ was incapable of binding this cationic dye (Figure 2). Compared to the WT control strain, no significant changes were observed in the ability of the *mnn41*Δ, *mnn43*Δ, *mnn44*Δ, *mnn45*Δ, and *mnn46*Δ null mutants to bind the dye. However, the *mnn42*Δ null mutant displayed a significant reduction in Alcian Blue binding, which was restored in the re-integrant control strain (Figure 2). Although it was not significant at *P* > 0.05, the *mnn47*Δ null mutant showed a reduction in the ability to bind the dye (*P* = 0.27). Since Figure 1 indicates Mnn41, Mnn42, and Mnn47 are closest paralogs to Mnn4, and because Mnn42 is required for full cell wall phosphomannosylation (Figure 2), we then generated a double null mutant lacking both *MNN41* and *MNN47*. This double mutant showed binding levels comparable to those found in the WT control strain, indicating *MNN42* was enough to sustain the normal cell wall phosphomannosylation (Figure 2). A triple *mnn41*Δ, *mnn42*Δ, and *mnn47*Δ null mutant was significantly attenuated in the ability to bind Alcian Blue compared to the WT control cells or the single *mnn42*Δ null mutant (Figure 2), indicating these family members may operate in tandem in cell wall phosphomannosylation. Complementation of this strain with *MNN41* did not increase Alcian Blue binding, but complementation with either *MNN42* or *MNN47*, resulted in a significant elevation in Alcian Blue binding in the triple null mutant (Figure 2), suggesting that *MNN42* and *MNN47* can play a significant role in the *C. albicans* cell wall phosphomannosylation. Mnn43, Mnn44, Mnn45, and Mnn46 belong to a different clade of the Mnn4 family (Figure 1). Therefore, we generated a quadruple null mutant lacking all four of these genes. However, this quadruple mutant did not show significant changes in the ability to bind Alcian Blue. The CRISPR-Cas9 single mutants reproduced the same Alcian Blue patterns generated by mini-Ura-Blaster disruption (data not shown). The CRISPR Cas9 generated octuple mutant, lacking all the *MNN4*-like genes and *MNN4* was unable to bind Alcian Blue, showing a phenotype similar to that observed in the *mnn4*Δ null mutant (Figure 2). These data indicate that some, but not all the members of the *MNN4*-like gene family participate in the *C. albicans* cell wall phosphomannosylation, and confirmed the dominant role of *MNN4* during phosphomannan synthesis in cells grown under standard laboratory conditions.

### Incremental increases in the gene dose of either *MNN41, MNN42, MNN46*, or *MNN47* partially complement the *C. albicans MNN4*Δ null mutant

**Figure 3:**
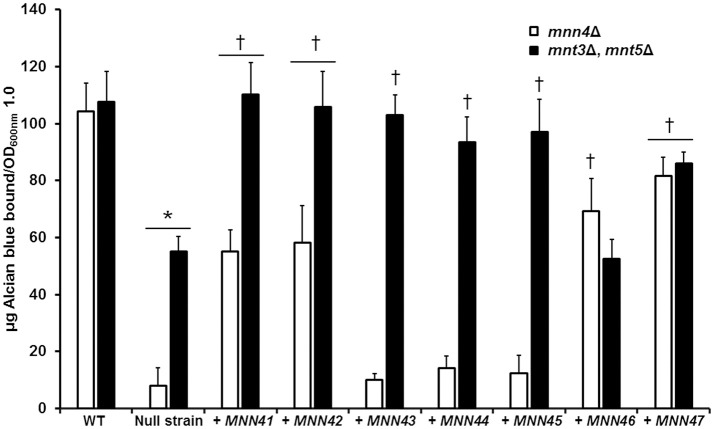
Incremental increases in the allele number of members of the *C. albicans MNN4*-like gene family restores the ability to bind Alcian Blue in mutants with defects in the phosphomannosylation. Null mutant cells lacking either *mnn4*Δ (open bars) or *mnt3*Δ, *mnt5*Δ (closed bars) were transformed with expression vectors containing each of the members of the *MNN4*-like gene family, then were grown in YPD medium, and ability to bind Alcian Blue was measured. The strains used are: NGY152 (WT), CDH15 (*mnn4*Δ), NGY1227 (*mnt3*Δ, *mnt5*Δ), see Table 1 for details of other strains. The data represent the means ± *SD* of three independent assays performed by triplicate. ^*^P < 0.05, compared with WT control cells. ^†^*P* < 0.05 when compared to the null mutant strain. The open bars correspond to strains in which the *mnn4*Δ null mutant was complemented with *MNN4* gene alleles. The closed bars refer to experiments in which the double *mnt3*Δ, *mnt5*Δ null mutant was complemented.

We next performed experiments in which transformed additional alleles of each member of the *MNN4*-like gene family into mutant strains lacking a functional *MNN4*, to assess whether this impacted on the ability to bind Alcian Blue. A Ura^−^ strain lacking *MNN4* (strain CDH7, see Table 1) was transformed with the *Stu*I-linearized CIp10 plasmid (Murad et al., 2000) harboring alleles of the *MNN4*-like gene family, generating a series of plasmids with an extra copy of each of these genes (see Table 1). All of these genes were expressed under the control of their own regulatory upstream sequences. We first analyzed whether the resulting increase in gene dosage impacted the expression levels of other *MNN4* genes. It was shown (Table 3) that in the *mnn4*Δ null mutant background the expression of other family members was not significantly affected with two exceptions - *MNN42* expression almost doubled and *MNN47* expression was increased by about 70%. All strains showed a significantly increased expression of the supplemented gene, when extra copies were transformed into the *mnn4*Δ null mutant background (Table 3). Therefore, incremental increases in the gene dose had a positive impact on the expression level of each member of the *MNN4*-like gene family. Next, we measured the ability of these strains to bind Alcian Blue and found that none of the complemented transformants were capable of restoring wild-type levels of Alcian Blue binding (Figure 3). However, the incremental increase in the gene dosage of *MNN41, MNN42, MNN46*, or *MNN47* resulted in strains that had significantly higher levels of dye bound, compared to the *mnn4*Δ null mutant (Figure 3). Therefore, increased expression of some, but all *MNN4*-like genes, was capable of partially complementing the *mnn4*Δ null mutant.

**Table 3:** Analysis of the expression of the members of the *C. albicans MNN4*-like gene family.

**Gene**	WT^*^	***mnn4*Δ^†^**	***mnn4*Δ + EV ^‡^**	***mnt3*Δ, *mnt5*Δ^¶^**	***mnt3*Δ, *mnt5*Δ + EV ^‡^**
*MNN41*	1.0 ± 0.01	1.1 ± 0.2	1.7 ± 0.1^††^	1.0 ± 0.09	1.8 ± 0.3^††^
*MNN42*	1.0 ± 0.01	1.9 ± 0.1^**^	2.5 ± 0.2^††^	1.1 ± 0.1	1.9 ± 0.1^††^
*MNN43*	1.0 ± 0.03	1.1 ± 0.2	1.8 ± 0.1^††^	1.0 ± 0.05	1.7 ± 0.06^††^
*MNN44*	1.0 ± 0.02	1.0 ± 0.1	2.0 ± 0.4^††^	1.1 ± 0.1	1.8 ± 0.1^††^
*MNN45*	1.0 ± 0.01	1.1 ± 0.3	1.9 ± 0.1^††^	1.1 ± 0.1	1.8 ± 0.09^††^
*MNN46*	1.0 ± 0.03	1.0 ± 0.2	1.9 ± 0.3^††^	1.0 ± 0.08	1.7 ± 0.1^††^
*MNN47*	1.0 ± 0.02	1.7 ± 0.2^**^	2.6 ± 0.3^††^	1.1 ± 0.1	1.9 ± 0.05^††^

* *Strain NGY152*.

† *Strain CDH15*.

‡ *EV, Expression vector, see Table 1 for strain details*.

¶ *Strain NGY1227*.

** *P < 0.05, when compared to the WT strain*.

†† *P < 0.05, when compared to the null mutant strain*.

### Increment in the gene dose of *MNN46* does not complement the *C. albicans mnt3*Δ, *mnt5*Δ null mutant

Using the same strategy, complementation experiments were performed using the double *mnt3*Δ, *mnt5*Δ null mutant background, which lacks the phosphomannosyltransferases required for 50% of the normal level of total cell wall phosphomannan (Mora-Montes et al., 2010). The Ura^−^ strain carrying this dual disruption was used as the parent for complementing transformations with all individual *MNN4*-like genes (Table 1). Expression analyses indicated that loss of both *MNT3* and *MNT5* did not affect the expression of other *MNN4*-like gene family members (Table 3). Upon insertion of an additional *MNN4* allele, the expression of the relevant family member was significantly increased (Table 3). When extra copies of the genes encoding *MNN41, MNN42, MNN43, MNN44, MNN45*, or *MNN47* were introduced in the double *mnt3*Δ, *mnt5*Δ null background, the resulting increased expression of each gene increased the ability of the double null mutant to bind Alcian Blue (Figure 3). However, increased *MNN46* expression in this double mutant did not affect Alcian Blue binding (Figure 3).

### Alterations in the gene dosage of *MNN4*-like genes affects the *C. albicans*-macrophage interaction

**Figure 4:**
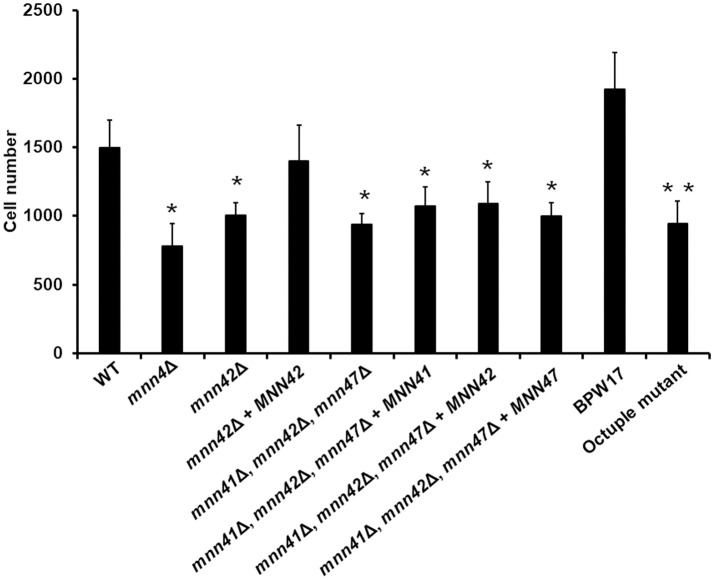
Phagocytosis of *C. albicans* null mutants lacking members of the *MNN4*-like gene family by RAW 264.7 macrophages. *C. albicans* yeast cells were labeled with Acridine Orange and incubated with RAW 264.7 macrophages at a MOI 3:1 for 2.5 h at 37°C under CO_2_. Macrophages were gated by FACS and 50,000 cells were counted/ sample. Results represent macrophages interacting with at least one fluorescent fungal cell. Strains used are: NGY152 (WT), CDH15 (*mnn4*Δ), HMY81 (*mnn42*Δ), HMY84 (*mnn42*Δ + *MNN42*), HMY198 (*mnn41*Δ, *mnn42*Δ, *mnn47*Δ), HMY199 (*mnn41*Δ, *mnn42*Δ, *mnn47*Δ + *MNN41*), HMY200 (*mnn41*Δ, *mnn42*Δ, *mnn47*Δ + *MNN42*), and HMY201 (*mnn41*Δ, *mnn42*Δ, *mnn47*Δ + *MNN47*). The data represent means ± *SD* for three independent assays performed by duplicate. ^*^P < 0.05 when compared against the WT strain. ^**^P < 0.05 when the octuple mutant was compared against the parental strain BWP17.

**Figure 5:**
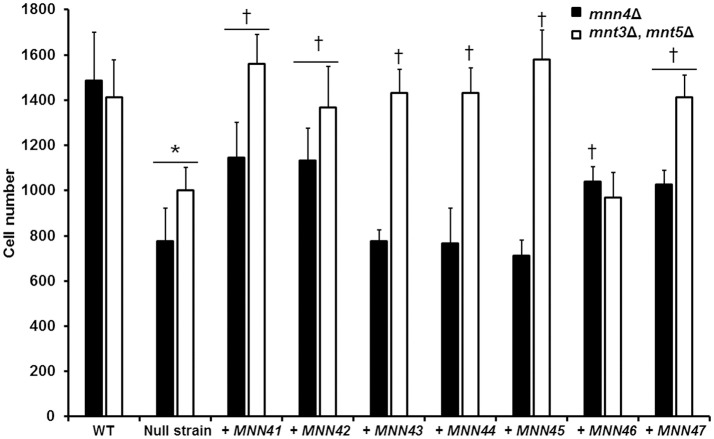
Transformation of the *mnn4* null mutant with additional alleles of the *C. albicans MNN4*-like gene family restores the *C. albicans*-RAW 264.7 macrophage interaction. Null mutant cells lacking either *mnn4*Δ (closed bars) or *mnt3*Δ, *mnt5*Δ (open bars) were transformed with expression vectors containing each of the members of the *MNN4*-like gene family. Cells were labeled with Acridine Orange and incubated with RAW 264.7 macrophages at a MOI 3:1 for 2.5 h at 37°C under a CO_2_ atmosphere. Then, macrophages were gated by FACS system and 50,000 cells were counted/sample. Results represent macrophages interacting with at least one fluorescent fungal cell. Strains used are: NGY152 (WT), CDH15 (*mnn4*Δ), NGY1227 (*mnt3*Δ, *mnt5*Δ), see Table 1 for details of the other strains. The data represent means ± *SD* of three independent biological replicates performed by duplicate. ^*^P < 0.05, compared with WT control cells. ^†^*P* < 0.05 is a comparison with the null mutant strain.

Because disruption of the cell wall phosphomannosylation is related to defective phagocytosis of *C. albicans* by macrophages (McKenzie et al., 2010), we then assessed whether gene disruption or gene supplementation of the *MNN4*-like gene family members impacted *C. albicans*-macrophages interactions. As reported previously (McKenzie et al., 2010), the *mnn4*Δ null mutant strain exhibited a 50% reduction in phagocytosis compared to WT control cells (Figures 4, 5). The *mnn42*Δ null mutant also showed a significant reduction in uptake by macrophages, that was restored to WT levels in the re-integrant control strain (Figure 4). The triple *mnn41*Δ, *mnn42*Δ, *mnn47*Δ null mutant also displayed a reduction in the phagocytosis when interacting with human macrophages, but reintroduction of a single disrupted gene failed to complement this phenotype (Figure 4). Although the parental strain BPW17 displayed increased phagocytosis compared to the NGY52 (WT) control strain (*P* = 0.0932, when compared WT and BPW17), the octuple null mutant showed a 50% reduction in the uptake by macrophages (Figure 4). The other null mutant strains generated in this work were phagocytosed to a similar extent to WT control cells (data dot shown).

When the interaction of macrophages and cells harboring an extra copy of the members of the *MNN4*-like gene family was analyzed, we observed that the cell uptake followed a similar trend to that described in the ability of binding Alcian Blue. Extra copies of either *MNN41, MNN42*, or *MNN46* in the *mnn4*Δ null background significantly increased yeast cell phagocytosis (Figure 5). Extra copies of all the family members, with the exception of *MNN46*, positively influenced the phagocytosis of cells in a genetic background lacking both *MNT3* and *MNT5* (Figure 5). Collectively, these data indicate the members of the *MNN4*-like gene family influence the *C. albicans*-human macrophage interaction and that phosphomannan content correlated positively with the degree of phagocytosis of the yeast cells by macrophages.

## Discussion

The study of the fungal cell wall of medically relevant organisms is of special interest, because most cell wall components contribute to the induced immune response, and are required for cell fitness and virulence (Díaz-Jiménez et al., 2012; Martinez-Alvarez et al., 2014; Netea et al., 2015). Such studies have the potential to unveil new molecular targets to expand the repertoire of drugs to treat fungal infections. Cell wall phosphomannosylation is a glycoprotein modification found only in a reduced group of yeast-like fungi, including *S. cerevisiae, Kloeckera brevis, Yarrowia lypolytica* and several species of the *Candida* genus, including *C. albicans* (Jigami and Odani, 1999; Butler et al., 2009; Gil et al., 2015). Although, in *C. albicans*, this cell wall modification is dispensable for cell viability, it participates in the interaction with effector molecules and cells of the immune response (Harris et al., 2009; McKenzie et al., 2010), and is a trait that varies between clinical isolates, with some showing reduced and others increased phosphomannan content of their cell walls (MacCallum et al., 2009). This suggests cell wall phosphomannosylation is part of the cellular phenotype that *C. albicans* can modify as it adapts to different environments.

The molecular machinery resulting in the synthesis of phosphomannan is more complex in *C. albicans* than that described in *S. cerevisiae*, where all the components of this biosynthetic process have been analyzed. This complexity in *C. albicans* is underlined by the presence of an extended *MNN4*-like gene family. None of the other *C. albicans MNN4*-like genes contains the lysine/glutamic acid repeats found in Mnn4, which is presumed to be required for phosphomannan production (Hobson et al., 2004). This may offer an explanation for the dominant role of Mnn4, since deletion of this gene was sufficient to eliminate Alcian Blue binding of cells grown under laboratory conditions. A conserved feature of Mnn4 and Mnn4-family proteins is the presence of the LicD domain. The *C. albicans* genome database identified only 8 encoded proteins containing this domain - Mnn4 and the seven *MNN4*-like family members. A similar analysis within the *S. cerevisiae* genome database identified only Mnn4 and Mnn14 as proteins containing this domain (data not shown). Although the functional role of this LicD domain in the phosphomannosylation mechanism remains unknown, we conclude this is a signature domain for the identification of proteins involved in the cell wall phosphomannosylation pathway in fungi. In *Ogataea minuta* (Akeboshi et al., 2009), *Yarrowia lipolytica* (Park et al., 2011), *Pichia pastoris* (Miura et al., 2004), *Candida parapsilosis*, and *Candida tropicalis* (our unpublished observations), the Mnn4-like proteins involved in the modification of glycoproteins with phosphomannan all contain the LiCD domain, providing additional support for this conclusion.

The null mutants generated here displayed no defects in morphology, ability to undergo yeast-hypha dimorphism and sensitivity to a range of cell wall perturbing agents. This is consistent with the phenotype reported for the *mnn4*Δ null mutant (Hobson et al., 2004). The only measured alteration in the phenotypes was the ability of cells to bind Alcian Blue and to interact with macrophages (Hobson et al., 2004; McKenzie et al., 2010). However, we would predict that any phenotype that depends on the net charge of the cell wall would likely to be affected, including the interaction with cationic peptides. Even though most of the single null mutants generated here did not show a marked reduction in the cell wall phosphomannan content, the subtle changes in phosphomannan content still affected the interaction with macrophages, indicating that, along with mannan and β1, 3-glucan (Heinsbroek et al., 2008), phosphomannan is a key cell wall component influencing immune recognition. Interestingly, although the cell wall phosphomannan content of the *mnn41, mnn42, mnn47* triple null mutant was partially restored by complementation with either *MNN42* or *MNN47*, this did not have a significant impact on the phagocytosis of the complemented strains. These data suggest that a minimal concentration of phosphomannan has to be present in the wall for optimal rates of phagocytosis by human macrophages. Since the full reduction of cell wall phosphomannan in the *mnn4*Δ null mutant did not affect virulence in the murine model of candidiasis (Hobson et al., 2004), we did not perform an exhaustive analysis of the virulence properties of this mutant collection.

The generation of an octuple mutant in *C. albicans* using the recently developed CRISPR-Cas9 system (Vyas et al., 2015) is one of the most extensive disruption protocols attempted in this fungus. To our knowledge, the disruption of a full gene family has been only reported for the *KRE2/MNT1* and *MNN2* gene families, which are composed of five and six members, respectively (Mora-Montes et al., 2010; Hall et al., 2013). The octuple mutant reported demonstrates the advantages that the CRISPR-Cas9 strategy can provide in the assessment of extended gene families in this organism.

We demonstrate that Mnn42, and to a lesser extent Mnn47, were the *MNN4* family members (other and Mnn4) that most affected cell wall phosphomannan synthesis. These genes were upregulated in a genetic background lacking *MNN4*. These data suggest that Mnn42 and Mnn47 contribute to canonical glycoprotein modification with mannosylphosphate, but that the presence of Mnn4 is obligately required to carry out this function. When the gene dose of *MNN4*-like gene family members was increased by transformation, we observed that overexpression of *MNN41, MNN42, MNN46* and *MNN47* partially restored the cell wall phosphomannan, indicating that these genes can compensate for the absence of Mnn4, only when their expression levels were increased. These findings support studies in *C. albicans* and *S. cerevisiae*, where strains that were heterozygous for the *MNN4* locus were unable to bind normal levels of Alcian Blue (Jigami and Odani, 1999; Hobson et al., 2004). We found that the transcript abundance was similar for *MNN4* and the *MNN4*-like gene family members in the WT control strain when cells were grown in YPD at 28°C (data not shown). Therefore, the dominant contribution of *MNN4* to phosphomannosylation over the members of the gene family may rely on posttranscriptional or posttranslational modifications that favor the accumulation of Mnn4 in the system. Analysis of the public available databases analyzed via transcriptomic microarrays revealed that *MNN4, MNN43*, and *MNN45* only undergo subtly altered levels of transcription when cells were grown under a variety of conditions, or exposed to different stressors (Enjalbert et al., 2003, 2006; Lorenz et al., 2004; Fradin et al., 2005; Karababa et al., 2006). However, expression of *MNN41* and *MNN42* was shown to be upregulated when the fungal cells interact with neutrophils (Fradin et al., 2005). The transcriptional regulation of the latter is also positively influenced when cells are grown in presence of 200 mM CaCl_2_ for 20, 40, or 60 min (Karababa et al., 2006). Expression of *MNN44* and *MNN46* was reported to be downregulated and upregulated, respectively, when cells were incubated under oxidative stress conditions (Enjalbert et al., 2003). Furthermore, *MNN46* expression was upregulated as a response to nitrogen or carbon starvation (Lorenz et al., 2004). Collectively, these results indicate the members of this gene family are transcriptionally regulated under a variety of growth conditions and that this regulation may influence the degree of glycoprotein phosphomannosylation. Moreover, it is not clear how members of this gene family are regulated in different micro niches during the natural history of an infection.

Overexpression of *MNN4*-like gene family members in a genetic background lacking Mnt3 and Mnt5 (Mora-Montes et al., 2010) also demonstrated that the gene dosage of any of the family members, with the exception of *MNN46*, restored the phosphomannan content to levels comparable to those found in the WT control cells. Again, this suggests that activation of phosphomannosyltransferase could be achieved by proteins that are solely activated by Mnn4, or proteins activated by the dual action of Mnn4 and other *MNN4*-like gene family members. This reinforces and extends previous reports studying the canonical Mnn4 protein (Hobson et al., 2004). This conclusion is inferred from the current report, where overexpression of most of the family members positively affected the phosphomannosylation thereby partially compensating for the lack of Mnn4. It is formally possible that all *MNN4*-like gene family members participate directly as phosphomannosyltransferases. Additional experiments using *in vitro* assays for this biochemical reaction would be required to address this possibility unequivocally.

The data reported here clearly demonstrated that *MNN4*-like genes have a significant role in *C. albicans* phagocytosis by macrophages; which contrast with previously reported results from our group, indicating that a *mnn4*Δ null mutant was readily phagocytosed as the WT control cells (Hobson et al., 2004). McKenzie et al. (2010) reported a comprehensive study of the role of protein mannosylation during phagocytosis and found that the experimental setting published in 2004 was overshadowing the differences between the WT and *mnn4*Δ strains. Hobson et al. (2004) used a yeast:macrophage ratio 20:1, for 1 h at 37°C, whereas McKenzie et al. (2010), and this report, used a yeast:macrophage ratio 3:1 and longer incubation times (3 and 2.5 h, respectively). These technical differences are likely to account for the apparent contradiction between our observations and those previously reported (Hobson et al., 2004).

In conclusion, we provide one of the most comprehensive analyses of an extended gene family to be attempted in any human pathogen. We provide evidence that the eight members of the *MNN4*-like gene family members all are capable of participating in the synthesis of cell wall phosphomannan in *C. albicans*, although the majority of the cell wall phosphomannosylation, under normal growth conditions, is achieved via Mnn4 itself. In addition, the observation that deletion of all individual members of this family influenced the ability of macrophages to phagocytose *C. albicans* cells suggests that this protein family collaborates to generate the charge in the cell wall, which in turn affects a number of immune recognition functions. Our data also underline the fact that the charge on the cell wall is critically important for the process of phagocytosis of this group of fungal pathogens.

## Author contributions

NG and HM conceived the study; RG, KJ, MH, ET, DC, AT, and BF performed the experiments; RG, KJ, MH, DD, ET, DC, AT, BF, NG, and HM analyzed the data; HM drafted the paper; RG, KJ, MH, DD, ET, DC, AT, BF, NG, and HM approved the final version of the manuscript.

### Conflict of interest statement

The authors declare that the research was conducted in the absence of any commercial or financial relationships that could be construed as a potential conflict of interest.
