# Supplementary material for: Phosphomannosylation and the Functional Analysis of the Extended Candida albicans MNN4-Like Gene Family
Source: Front Microbiol. 2017 Nov 6;8:2156. doi: 10.3389/fmicb.2017.02156 (PMC5681524; doi:10.3389/fmicb.2017.02156)
Supplement: Supplementary file 1 [file DataSheet1.docx]

Supplementary Material

**Phosphomannosylation and the functional analysis of the extended *Candida albicans* *MNN4*-like gene family**

Roberto J. González-Hernández^1,*^, Kai Jin^2,3*^, Marco J. Hernández-Chávez^1^, Diana F. Díaz-Jiménez^4^, Elías Trujillo-Esquivel^1^, Diana M. Clavijo-Giraldo^1^, Alma K. Tamez-Castrellón^1^, Bernardo Franco^1^, Neil A. R, Gow^2,†^ , and Héctor Manuel Mora-Montes^1,†^

^1^Departamento de Biología, División de Ciencias Naturales y Exactas, Campus Guanajuato, Universidad de Guanajuato, Noria Alta s/n, col. Noria Alta, C.P. 36050, Guanajuato, Gto., México.

^2^Aberdeen Fungal Group, Institute of Medical Sciences, Foresterhill, University of Aberdeen, AB25 2ZD, Aberdeen, UK.

^3^School of Life Sciences, Chongqing University, 55# Daxuecheng Southern Road, Shapingba District, Chongqing 4001331, People’s Republic of China.

^4^Centro de Investigaciones y de Estudios Avanzados del IPN, Apartado Postal 629, 36500 Irapuato, Guanajuato, México.

*****Both authors contributed equally to this work

^†^**Correspondence:** Corresponding Authors, Neil A. R. Gow, ^2^Aberdeen Fungal Group, Institute of Medical Sciences, Foresterhill, University of Aberdeen, AB25 2ZD, Aberdeen, UK. **Email:** [n.gow@abdn.ac.uk](mailto:n.gow@abdn.ac.uk); Héctor M Mora-Montes: Departamento de Biología, División de Ciencias Naturales y Exactas, Campus Guanajuato, Universidad de Guanajuato, Noria Alta s/n, col. Noria Alta, C.P. 36050, Guanajuato, Gto., México. **Email:** [hmora@ugto.mx](mailto:hmora@ugto.mx)

# Supplementary Data

| **Table S1. Oligonucleotides used for disruption of the *MNN4*-like gene family members using the mini-Ura-blaster technique** | |  |
| --- | --- | --- |
| **Sequence** | **Targeted gene** | |
| 5´GAATTTGGCAACACTGTTCCCAATATATATATAATCAACACCATACACTCATACCACAATATACTACAAATGTGGAATTGTGAGCGGATA | Forward primer for *MNN41* (C2_03710W_A)* | |
| 5’GCAATAATCTAATGCTATTTATATATATATATGTGTGTGTGAATATACCTAAACGTCTAGATAGGTAAAA GTTTTCCCAGTCACGACGTT | Reverse primer for *MNN41* (C2_03710W_A)* | |
| 5’ATGTTCATTATTAGAAGATCTCGAGGAATACTATTATTGGTTTCAATTGTTGTTTTCAATTTGATTGTATTGTGGAATTGTGAGCGGATA | Forward primer for *MNN42* (C2_03690C_A)* | |
| 5’TCAGTTAATCTCATTAACAATTTCATTTCTATTTAATGGATCATCGTTATCACCACTACTATTTTTGCTGGTTTTCCCAGTCACGACGTT | Reverse primer for *MNN42* (C2_03690C_A)* | |
| 5’ATTATACTGACCATTACTCTGTGTTTTGGACTAATTATTCACAAGGAAACCAAAAAAACCTACACTAGTTTGTGGAATTGTGAGCGGATA | Forward primer for *MNN43* (C1_02670C_A)* | |
| 5’TCCAATAACGCTATTTAAAGTTGCTGTCTAGTTCCAAATTTTTTTCTAAACCAATCAACACATTAAGAATGTTTTCCCAGTCACGACGTT | Reverse primer for *MNN43* (C1_02670C_A)* | |
| 5’TAGTCAATAAATGGAATATCAATACAGTAGCTGATCAATCCAGTTCTTTCGTTTACAGTCTACTATATACTGTGGAATTGTGAGCGGATA | Forward primer for *MNN44* (C1_02680C_A)* | |
| AAATATTTTAATTTCTCTATAGCCACTCTATGTAAAGATAAGAAAAACAATGGTAATAGCCATCTAGTATGTTTTCCCAGTCACGACGTT | Reverse primer for *MNN44* (C1_02680C_A)* | |
| 5’TTTTGATTATACTTCTTTCTTTACTTGAATATTGATTTATATCAACCGTTGAAGGTGCCATTTTATAACCTTGTGGAATTGTGAGCGGATA | Forward primer for *MNN45* (C6_02830W_A)* | |
| 5’GTTCACAGGTATATAAAATAGAGGGAAGTGTATCAAAGGTGGAAAAAAAATAGACATTTCCGTGTCTCCCGTTTTCCCAGTCACGACGTT | Reverse primer for *MNN45* (C6_02830W_A)* | |
| 5’CTTCAAGGATTGAATCAGAATCAACACTTAGAAGGAGCAATCATTTGCCCATTACTATCTTTGACTTGACTGTGGAATTGTGAGCGGATA | Forward primer for *MNN46* (C4_06990W_A)* | |
| 5’AAAAAAATATTTTAGAAATCTAAAAACACTAAATATATTGAAATGTTATAGAATTTATCTTGTTATGCATGTTTTCCCAGTCACGACGTT3’ | Reverse primer for *MNN46* (C4_06990W_A)* | |
| 5’TCCACATTCTACTGATCAAGTCATATTCCTTTACCCTATCCTTTCTATATTCATTGATATATTATTTATATGTGGAATTGTGAGCGGATA | Forward primer for *MNN47* (C1_09130W_A)* | |
| 5’TAATTAATTAATTAATAAAATAGTACATTAACTATATCTATAAATATAATTAAAACAAAAATAGAAAACTGTTTTCCCAGTCACGACGTT | Reverse primer for *MNN47* (C1_09130W_A)* | |

The underline bases correspond to complementary sequences to pDDB57 plasmid

*Systematic name at <http://www.candidagenome.org>

| **Table S2. Oligonucleotides used for disruption of the *MNN4*-like gene family members using the CRISP-Cas9 system** | | |
| --- | --- | --- |
| Primer | Sequence^a^ | Remarks |
| Mnn4-F | 5’ATTTGTCCAATTAGCATTAATATCAG | Primer pair used to clone sgRNA |
| Mnn4-R | 5’AAAACTGATATTAATGCTAATTGGAC |  |
| Mnn41-F | 5’ATTTGTTAATCTTCAATTCAATCAAG | Primers used to clone sgRNA |
| Mnn41-R | 5’AAAACTTGATTGAATTGAAGATTAAC |  |
| Mnn42-F | 5’ATTTGTGGAGTTTGAGCAATGATGCG | Primers used to clone sgRNA |
| Mnn42-R | 5’AAAACGCATCATTGCTCAAACTCCAC |  |
| Mnn43-F | 5’ATTTGAGAAGAAGATTCACTCCCTGG | Primers used to clone sgRNA |
| Mnn43-R | 5’AAAACCAGGGAGTGAATCTTCTTCTC |  |
| Mnn44-F | 5’ATTTGAGAATTGACAAGTCATAAAGG | Primers used to clone sgRNA |
| Mnn44-R | 5’AAAACCTTTATGACTTGTCAATTCTC |  |
| Mnn45-F | 5’ATTTGTATGACCAATACCGATTTGCG | Primers used to clone sgRNA |
| Mnn45-R | 5’AAAACGCAAATCGGTATTGGTCATAC |  |
| Mnn46-F | 5’ATTTGATCTACAATTTGACAAGGATG | Primers used to clone sgRNA |
| Mnn46-R | 5’AAAACATCCTTGTCAAATTGTAGATC |  |
| Mnn47-F | 5’ATTTGGTTGATAAAGTATCTACCATG | Primers used to clone sgRNA |
| Mnn47-R | 5’AAAACATGGTAGATACTTTATCAACC |  |
| Mnn4-Top | 5’GAATTTTC*AGATCT*AATATTTCTGGCTAGAAGGAAAAATTTCCAATTAGCATTAATATCA | Primers used to clone repair template. Italicized sequence is a restriction sites for BglII |
| Mnn4-Bottom | 5’TAGTTGATAGTATGACAAATGAACCAAAAAATAATAATGCTGATATTAATGCTAATTGGA |  |
| Mnn41-Top | 5’CCAAACTAAATATATAGACAAACCA*ACTAGT*ATCAAAAGATTAATCTTCAATTCAATCAA | Primers used to clone repair template. Italicized sequence is a restriction sites for SpeI |
| Mnn41-Bottom | 5’AAAACAAATTATAATCATAAACTAAGATATTATAATATGATTGATTGAATTGAAGATTAA |  |
| Mnn42-Top | 5’AAATTACCAACCAGATTCCCCTTGATC*ACTAGT*TAGTCGTGGAGTTTGAGCAATGATGC | Primers used to clone repair template. Italicized sequence is a restriction sites for SpeI |
| Mnn42-Bottom | 5’TGCAATTTCTTTATCATGATTCATAGATAATGCAAATTGTGCATCATTGCTCAAACTCCA |  |
| Mnn43-Top | 5’ACTATTACAACGACGATC*AGATCT*CCATCGGTTAACTCAAGAAGAAGATTCACTCCCTG | Primers used to clone repair template. Italicized sequence is a restriction sites for BglII |
| Mnn43-Bottom | 5’CGTTTGACTTTGTGAAGCAACTGTAATAGTTCTTGTTTGTCAGGGAGTGAATCTTCTTCT |  |
| Mnn44-Top | 5’AGTGGACTTCAATTAGGATAA*AGATCT*GGTTGAAAAGCCAAGAATTGACAAGTCATAAAG | Primers used to clone repair template. Italicized sequence is a restriction sites for BglII |
| Mnn44-Bottom | 5’TCCCACATTTCTTGAGTAAACTCATTTACATGAACCTTGACTTTATGACTTGTCAATTCT |  |
| Mnn45-Top | 5’CTAAATTGAATTGAGTCAAAA*AGATCT*AGGCAAATATTATATGACCAATACCGATTTGC | Primers used to clone repair template. Italicized sequence is a restriction sites for BglII |
| Mnn45-Bottom | 5’TTCAAAAATTGTTTGATTGGGACTTTGATTTTAGTTTTGAGCAAATCGGTATTGGTCATA |  |
| Mnn46-Top | 5’GAAAAACGTCTCAAGTGATTG*GATATC*TCAACCATATTCATCTACAATTTGACAAGGAT | Primers used to clone repair template. Italicized sequence is a restriction sites for EcoRV |
| Mnn46-Bottom | 5’CGCAGCCAAAGTAACGGGTGTTTTCTCTACACAATTCTGTATCCTTGTCAAATTGTAGAT |  |
| Mnn47-Top | 5’TACTTTAAA*AGATCT*TTAACACAGCAATCCAAATATCAAGTTGATAAAGTATCTACCAT | Primers used to clone repair template. Italicized sequence is a restriction sites for BglII |
| Mnn47-Bottom | 5’CCAATCATCCCAATGGAAATAAACTCCATGGGAATCATGAATGGTAGATACTTTATCAAC |  |
| Mnn4-VF | 5’CCACCACTTCCTACACACCA | Primers used to amplify the flanking region, and the PCR product digested with BglII, whose site was introduced during the mutagenesis. |
| Mnn4-VR | 5’ATCGTGATGGGCGGTTGTCA |  |
| Mnn41-VF | 5’TCAATTCCAATTCTAATCTACG | Primers used to amplify the flanking region, and the PCR product digested with SpeI, whose site was introduced during the mutagenesis. |
| Mnn41-VR | 5’CAACCCAATCAGACCAAT |  |
| Mnn42-VF | 5’TAGAAGATCTCGAGGAATAC | Primers used to amplify the flanking region, and the PCR product digested with SpeI, whose site was introduced during the mutagenesis. |
| Mnn42-VR | 5’GTTCTTGATTAGTGGTTGGT |  |
| Mnn43-VF | 5’ATTCACAAGGAAACCAAA | Primers used to amplify the flanking region, and the PCR product digested with BglII, whose site was introduced during the mutagenesis. |
| Mnn43-VR | 5’AATGGAATGTTGAAGGGT |  |
| Mnn44-VF | 5’ACCCACAACAACAACAAA | Primers used to amplify the flanking region, and the PCR product digested with BglII, whose site was introduced during the mutagenesis. |
| Mnn44-VR | 5’CTGCATAACCTGGAACAT |  |
| Mnn45-VF | 5’AACTAACTGGCCCATACC | Primers used to amplify the flanking region, and the PCR product digested with BglII, whose site was introduced during the mutagenesis. |
| Mnn45-VR | 5’AATCAATCCAATCAACCC |  |
| Mnn46-VF | 5’TAAATACTCCAGCATCAG | Primers used to amplify the flanking region, and the PCR product digested with EcoRV, whose site was introduced during the mutagenesis. |
| Mnn46-VR | 5’CATTACCCACAGTCATAG |  |
| Mnn47-VF | 5’TACATTTGCAGCAAGAAG | Primers used to amplify the flanking region, and the PCR product digested with BglII, whose site was introduced during the mutagenesis. |
| Mnn47-VR | 5’ATCAGTAAATACCCAAGC |  |

| **Table S3. Oligonucleotides used for complementation with members of the *MNN4*-like gene family** | |  |
| --- | --- | --- |
| **Sequence** | **Targeted gene** | |
| 5’GCGGCCGCTTAGATTAGACCACACGAAAAA | Forward primer for *MNN41* (C2_03710W_A)* | |
| 5’GCGGCCGCGCAACTAATATTATCCATCAATTTC | Reverse primer for *MNN41* (C2_03710W_A)* | |
| 5´GCGGCCGCGGTGCATACGTGTTCAGAAA | Forward primer for *MNN42* (C2_03690C_A)* | |
| 5´GCGGCCGCATGTTTTGGAGGAGGAGGAT | Reverse primer for *MNN42* (C2_03690C_A)* | |
| 5’GCGGCCGCCGACAATGCCAGAATATGTG | Forward primer for *MNN43* (C1_02670C_A)* | |
| 5’GCGGCCGCAAGAAGTAGTGGATTTTACTGGA | Reverse primer for *MNN43* (C1_02670C_A)* | |
| 5’GCGGCCGCGTGAATTATTGCCTCACGCTG | Forward primer for *MNN44* (C1_02680C_A)* | |
| 5’GCGGCCGCGGGAAATGATCAATGCAAG | Reverse primer for *MNN44* (C1_02680C_A)* | |
| 5´GCGGCCGCTCGTGTGCGACCTAACCTG | Forward primer for *MNN45* (C6_02830W_A)* | |
| 5’GCGGCCGCCAACGTCAACGAACCCAG | Reverse primer for *MNN45* (C6_02830W_A)* | |
| 5’GCGGCCGCCCAACTATGCTCCGGTGG | Forward primer for *MNN46* (C4_06990W_A)* | |
| 5’GCGGCCGCGGTTGGTGGATTGATTCATTG | Reverse primer for *MNN46* (C4_06990W_A)* | |
| 5’GCGGCCGCTGTACGGCTATAAACTCCCC | Forward primer for *MNN47* (C1_09130W_A)* | |
| 5’GCGGCCGCGTGGCAAGCAAGACGAG | Reverse primer for *MNN47* (C1_09130W_A)* | |

The underline bases correspond to bases added to create a Not I site.

*Systematic name at <http://www.candidagenome.org>

| **Table S4. Oligonucleotides used in gene expression assays by RT-qPCR** | |  |
| --- | --- | --- |
| **Sequence** | **Targeted gene** | |
| 5’GGTGGAGAATGCTGGGACTA | Forward primer for *MNN41* (C2_03710W_A)* | |
| 5’GGTCAAGGCTAACCCAGTGA | Reverse primer for *MNN41* (C2_03710W_A)* | |
| 5´TTCGTGGAGTTTGAGCAATG | Forward primer for *MNN42* (C2_03690C_A)* | |
| 5´GCCCAATGTGAATCTTGGAT | Reverse primer for *MNN42* (C2_03690C_A)* | |
| 5’TCCCTTGAGGTATTCCATGC | Forward primer for *MNN43* (C1_02670C_A)* | |
| 5’TGTCGAGCCTCATCTGTCAC | Reverse primer for *MNN43* (C1_02670C_A)* | |
| 5’ACCGAGGCTCAACATAATGG | Forward primer for *MNN44* (C1_02680C_A)* | |
| 5’GCACATAAACAGGCGTTCCT | Reverse primer for *MNN44* (C1_02680C_A)* | |
| 5´GGCGAAACTGTCGAACAAAT | Forward primer for *MNN45* (C6_02830W_A)* | |
| 5’TGCATCTTGCTGTTGTTGGT | Reverse primer for *MNN45* (C6_02830W_A)* | |
| 5’GAAGACTCGGGATGGCATTA | Forward primer for *MNN46* (C4_06990W_A)* | |
| 5’GAAAGATGGCTCCATTCCAA | Reverse primer for *MNN46* (C4_06990W_A)* | |
| 5’TGGGATGATTGGGTTGATTT | Forward primer for *MNN47* (C1_09130W_A)* | |
| 5’GGCATCGACCTCGAATATGT | Reverse primer for *MNN47* (C1_09130W_A)* | |
| 5’GGAATCCGTTGGTGTTGAAG | Forward primer for *RPP2B* (C3_04680W_A)* | |
| 5’CGGATGGGACAGAAGCTAAT | Reverse primer for *RPP2B* (C3_04680W_A)* | |

*Systematic name at <http://www.candidagenome.org>
